# Prevention of coronary obstruction in patients at risk undergoing transcatheter aortic valve implantation: the Hamburg BASILICA experience

**DOI:** 10.1007/s00392-021-01881-4

**Published:** 2021-06-22

**Authors:** Dirk Westermann, Sebastian Ludwig, Daniel Kalbacher, Clemens Spink, Matthias Linder, Oliver D. Bhadra, Julius Nikorowitsch, Lara Waldschmidt, Till Demal, Lisa Voigtländer, Andreas Schaefer, Moritz Seiffert, Simon Pecha, Niklas Schofer, Adam B. Greenbaum, Hermann Reichenspurner, Stefan Blankenberg, Lenard Conradi, Johannes Schirmer

**Affiliations:** 1 Department of Cardiology, University Heart and Vascular Center Hamburg, University Medical Center Hamburg-Eppendorf, Martinistrasse 52, 20246 Hamburg, Germany; 2 German Center for Cardiovascular Research (DZHK), Partner Site Hamburg/Luebeck/Kiel, Hamburg, Germany; 3 Department of Diagnostic and Interventional Radiology and Nuclear Medicine, University Medical Center Hamburg-Eppendorf, Hamburg, Germany; 4 Department of Cardiovascular Surgery, University Heart and Vascular Center Hamburg, Hamburg, Germany; 5 Structural Heart and Valve Center, Emory University Hospital, Atlanta, GA USA

**Keywords:** Transcatheter aortic valve implantation, Coronary obstruction, BASILICA

## Abstract

**Objectives:**

This study aimed to assess the clinical outcome of the bioprosthetic or native aortic scallop intentional laceration to prevent iatrogenic coronary obstruction (BASILICA) technique in a single-center patient cohort considered at high or prohibitive risk of transcatheter aortic valve implantation (TAVI)-induced coronary obstruction.

**Methods:**

Between October 2019 and January 2021, a total of 15 consecutive patients (age 81.0 [78.1, 84.4] years; 53.3% female; EuroSCORE II 10.6 [6.3, 14.8] %) underwent BASILICA procedure prior to TAVI at our institution. Indications for TAVI were degeneration of stented (*n* = 12, 80.0%) or stentless (*n* = 1, 6.7%) bioprosthetic aortic valves, or calcific stenosis of native aortic valves (*n* = 2, 13.3%), respectively. Individual risk of TAVI-induced coronary obstruction was assessed by pre-procedural computed tomography analysis. Procedural and 30-day outcomes were documented in accordance with Valve Academic Research Consortium (VARC)-2 criteria.

**Results:**

BASILICA was attempted for single left coronary cusp in 12 patients (80.0%), for single right coronary cusp in 2 patients (13.3%), and for both cusps in 1 patient (6.7%), respectively. The procedure was feasible in 13 patients (86.7%) resulting in effective prevention of coronary obstruction, whilst TAVI was performed without prior successful bioprosthetic leaflet laceration in two patients (13.3%). In one of these patients (6.7%), additional chimney stenting immediately after TAVI was performed. No all-cause deaths or strokes were documented after 30 days.

**Conclusion:**

The BASILICA technique appears to be a feasible, safe and effective concept to avoid iatrogenic coronary artery obstruction during TAVI in both native and bioprosthetic valves of patients at high or prohibitive risk.

**ClinicalTrials.gov Identifier:** NCT04227002 (Hamburg AoRtic Valve cOhoRt).

**Supplementary Information:**

The online version contains supplementary material available at 10.1007/s00392-021-01881-4.

## Introduction

Over the past decade, transcatheter aortic valve implantation (TAVI) has evolved to routine therapy for patients suffering from symptomatic aortic valve disease [1]. However, acute coronary obstruction during TAVI, either directly or indirectly by sequestering the sinus of Valsalva (SOV) at the sinotubular junction (STJ), is a rare but potentially devastating complication. Occurring in < 1% of TAVI procedures, high mortality rates of up to 50% within 30 days have been reported leading to increasing efforts to anticipate and prevent this life-threatening complication [2–4]. Risk of coronary obstruction is particularly elevated in patients with prior surgical aortic valve replacement (SAVR) undergoing valve-in-valve (ViV) TAVI, with a four- to sixfold higher incidence compared to TAVI for native aortic valve disease [2, 4, 5]. Pre-procedural contrast-enhanced multislice computed tomography (MSCT) imaging with virtual transcatheter heart valve (THV) implantation plays a pivotal role in identifying these patients at high or prohibitive risk [2, 6].

Until recently, protective management in patients with increased risk of coronary obstruction during TAVI included upfront coronary wire advancement in the threatened coronary artery with the option to perform immediate chimney stenting [7]. Bioprosthetic or native aortic scallop intentional laceration to prevent iatrogenic coronary artery obstruction (BASILICA) technique is an alternative and promising endovascular electrosurgical method to avoid fatal coronary occlusion [8]. Initial results have demonstrated feasibility and safety of this procedure [9, 10]. With this study, we present a single-center experience of 15 patients with either native aortic stenosis (AS) or degenerated surgical bioprostheses, all at high risk of coronary obstruction after native valve TAVI or ViV TAVI, undergoing intentional leaflet laceration with the BASILICA technique.

## Methods

### Study population and data acquisition

**Fig. 1:**
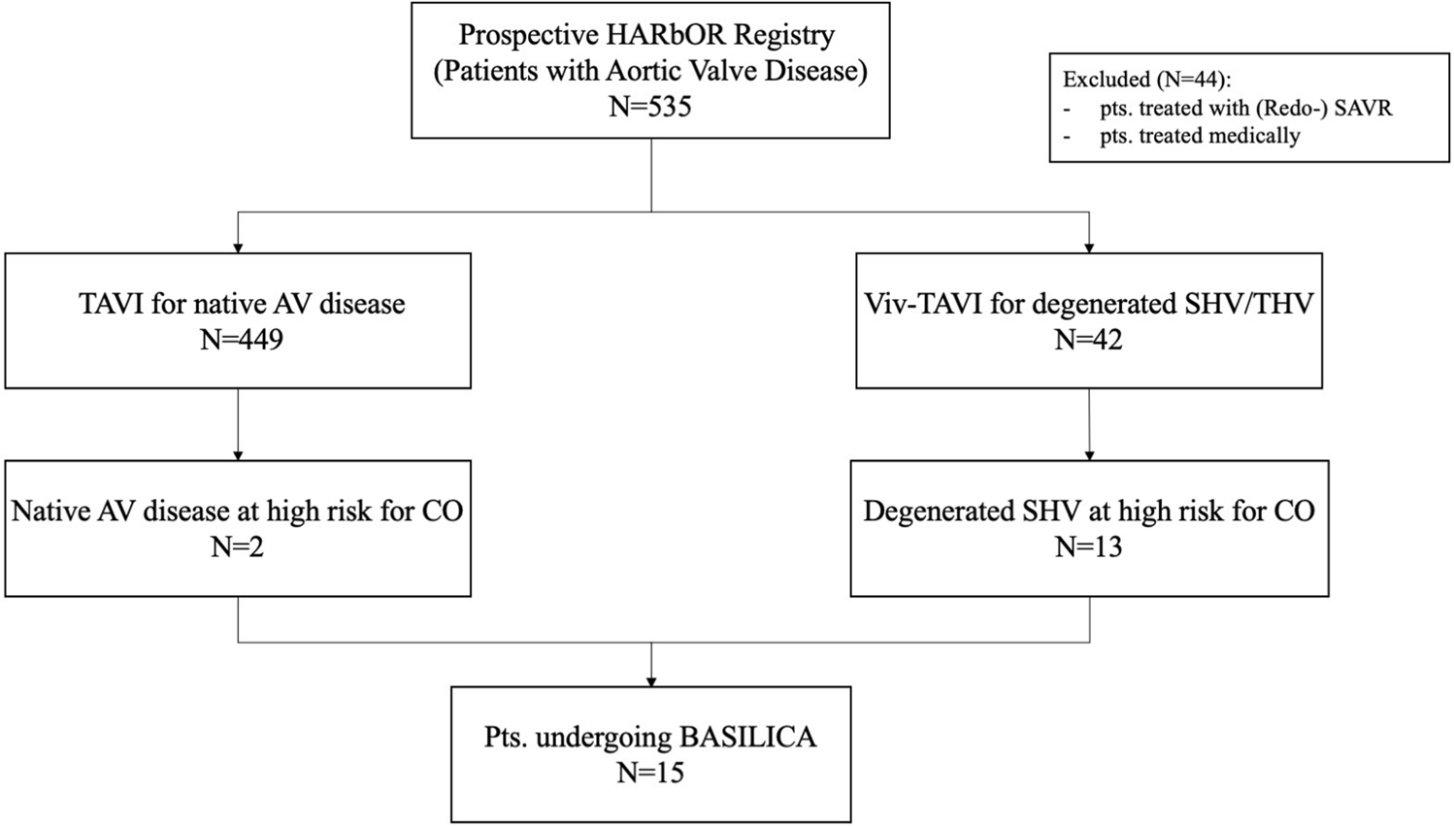
Study flowchart. *AR* aortic regurgitation, *AS* aortic stenosis, *BASILICA* bioprosthetic or native aortic scallop intentional laceration to prevent iatrogenic coronary artery obstruction, *CO* coronary obstruction, *SAVR* surgical aortic valve replacement, *SHV* surgical heart valve, *TAVI* transcatheter aortic valve implantation, *THV* transcatheter heart valve, *ViV* valve-in-valve

Since its initiation at University Heart and Vascular Center Hamburg in 2019, the prospective Hamburg AorRtic valve cOhoRt (HARbOR) Registry (NCT04227002) has included 535 patients with aortic valve disease. Of these, 449 patients were treated with TAVI for symptomatic native aortic valvular disease, whilst 42 patients underwent ViV TAVI for degenerated bioprosthetic aortic valves. Patients treated medically or with (Redo-)SAVR (*N* = 44) were excluded from this study. Between October 2019 and January 2021, a total of 15 patients at high or prohibitive risk of coronary obstruction were identified as candidates for the BASILICA procedure, of whom 2 patients (13.3%) received native valve TAVI and 13 patients (86.7%) underwent ViV TAVI. A study flowchart is given in Fig. 1. Clinical endpoints were adjudicated according to current Valve Academic Research Consortium (VARC)-2 criteria after 30 days. Allocation of patients to TAVI followed the consensus of the local dedicated interdisciplinary heart team based on established criteria. All patients provided informed consent to the procedure and data acquisition. The study was approved by the local ethics committee and was conducted in accordance with the Declaration of Helsinki.

### Echocardiograhpic measurements

Transthoracic and transesophageal echocardiography at baseline were performed in all patients. Severity of AS and grade of bioprosthetic valve degeneration were assessed according to current ESC/EACTS guidelines for the management of valvular heart disease [11]. Evaluation of THV function at discharge was assessed by transthoracic echocardiography.

### Computed tomography analysis

All patients underwent pre-procedural contrast-enhanced MSCT. Three-dimensional reconstruction of the aortic valve and risk assessment for coronary obstruction were performed using a dedicated software (3mensio Structural Heart V9.1, Pie Medical Imaging, Maastricht, The Netherlands). The annular plane was defined using a 3-point technique [12]. Valve-to-coronary (VTC) and valve-to-sinotubular junction (VTSTJ) distances were measured following virtual implantation of the THV planned for each individual procedure, based on a previously published method [13]. Coronary height [left coronary artery (LCA); right coronary artery (RCA)] and STJ height were measured orthogonally from the pre-defined aortic valve annulus (or surgical prosthesis). SOV diameters were assessed separately for each cusp, while STJ diameter was derived from STJ perimeter.

### Concept of Deficient and Sequestered Sinus

**Fig. 2:**
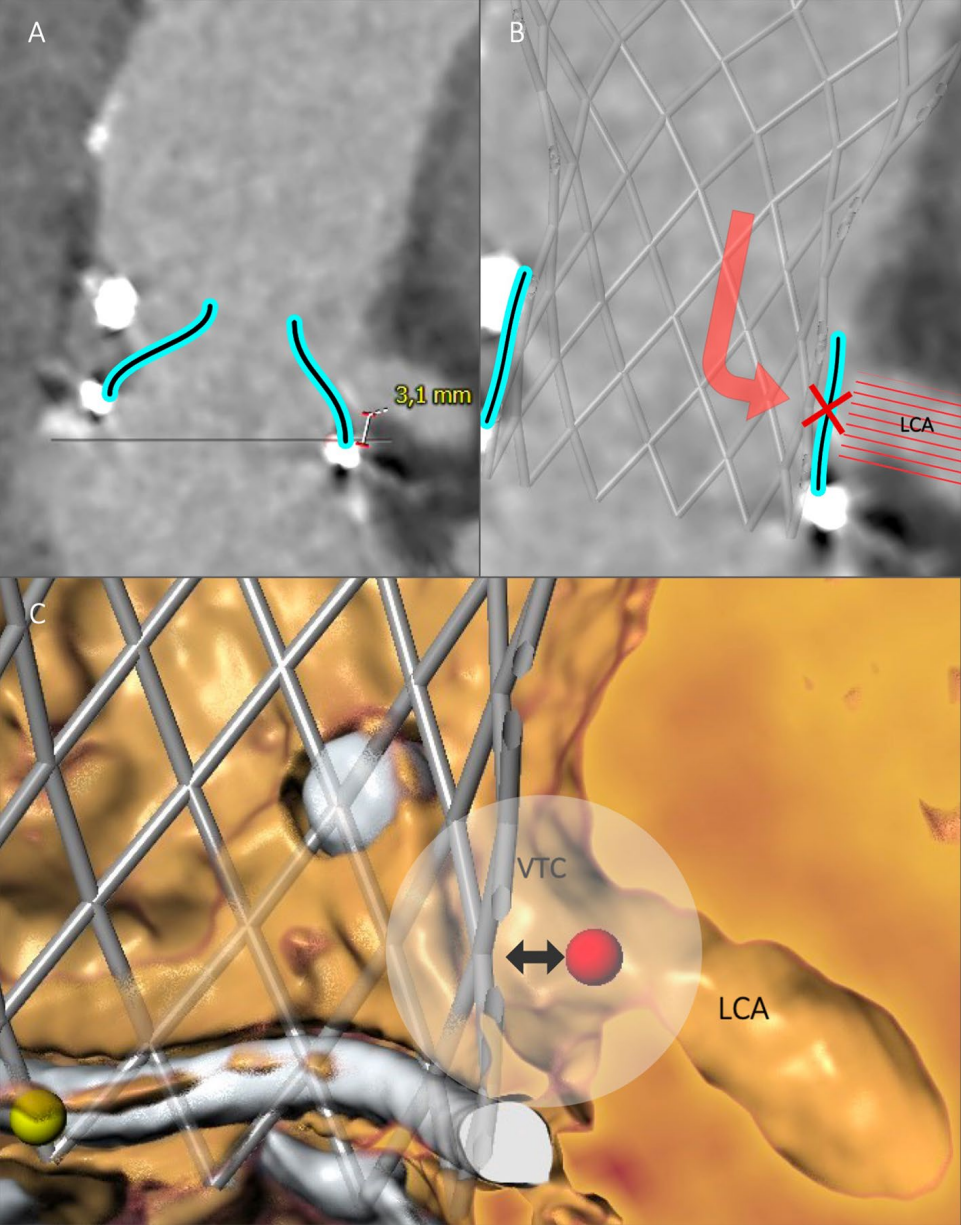
Deficient Sinus. 3mensio Structural Heart V9.1, Pie Medical Imaging, Maastricht, Netherlands. **A** Deficient Sinus (pre-procedural CT). Deficient Sinus is defined by low coronary height and narrow sinus of Valsalva. Measurement (3.1 mm) indicates low left coronary artery height. Surgical valve leaflets have been drawn retrospectively. **B** Coronary obstruction in a patient with Deficient Sinus [pre-procedural CT with a virtually implanted THV (Evolut R, *Medtronic*)]. Following THV implantation, the surgical valve leaflet directly obstructs the complete sinus of Valsalva, including the LCA, inhibiting coronary blood flow. **C** Valve-to-coronary (VTC) distance (3D CT reconstruction). *CT* computed tomography, *LCA* left coronary artery, *THV* transcatheter heart valve, *VTC* valve-to-coronary distance

**Fig. 3:**
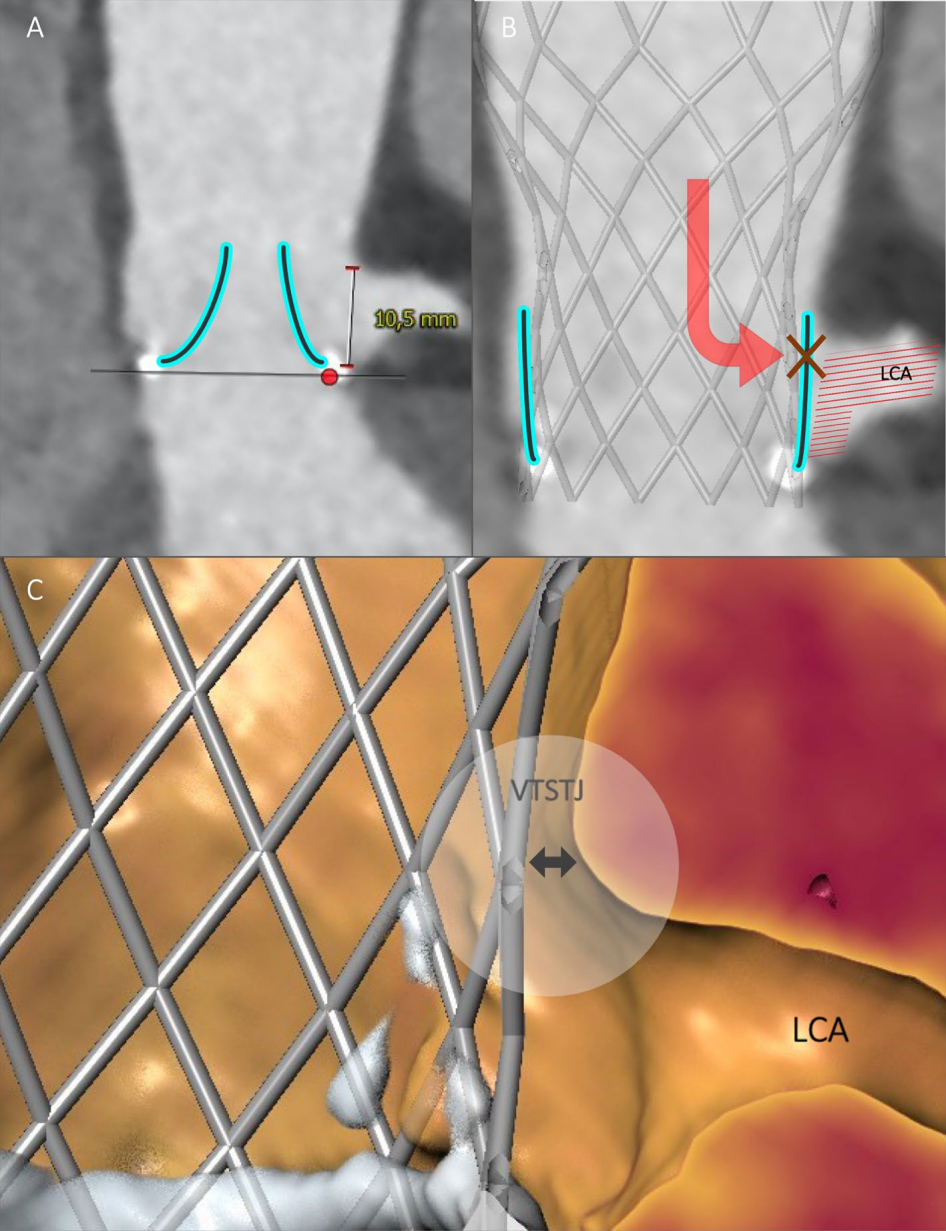
Sequestered Sinus. **A** Sequestered Sinus (pre-procedural CT). Sequestered Sinus is defined by low STJ height (indicated measurement, 10.5 mm) and narrow STJ diameter. Surgical valve leaflets have been drawn in retrospectively. **B** Coronary obstruction in a patient with Sequestered Sinus [pre-procedural CT with a virtually implanted THV (Evolut R, *Medtronic*)]. Following THV implantation the surgical valve leaflet indirectly inhibits blood flow to SOV and LCA by obstruction at the STJ. **C** Valve-to-sinotubular junction (VTSTJ) distance (3D CT reconstruction). *CT* computed tomography, *LCA* left coronary artery, *SOV* sinus of Valsalva, *STJ* sinotubular junction, *THV* transcatheter heart valve, *VTSTJ* valve-to-sinotubular junction

Indication for BASILICA was assessed according to the concept of *Deficient* and *Sequestered Sinus*, as described by Lederman et al. [6], leading to high or prohibitive risk of either direct or indirect coronary obstruction, respectively. Patients with *Deficient Sinus* (Fig. 2) are characterized by small SOV diameter and low coronary artery height. If left untreated, implantation of a THV may result in direct coronary obstruction by a leaflet due to an obliterated or effaced SOV in these patients. The VTC distance is considered the main determinant for risk of coronary obstruction in patients with *Deficient Sinus*. In patients with *Sequestered Sinus* (Fig. 3), multiple factors may result in high risk of indirect coronary obstruction. Depending on STJ height, STJ diameter and outer THV frame diameter, blood flow into the SOV may be impaired by leaflet obstruction on the height of STJ. Risk of coronary obstruction in patients with *Sequestered Sinus* is assessed by measurement of VTSTJ distance [6].

### BASILICA procedure

**Fig. 4:**
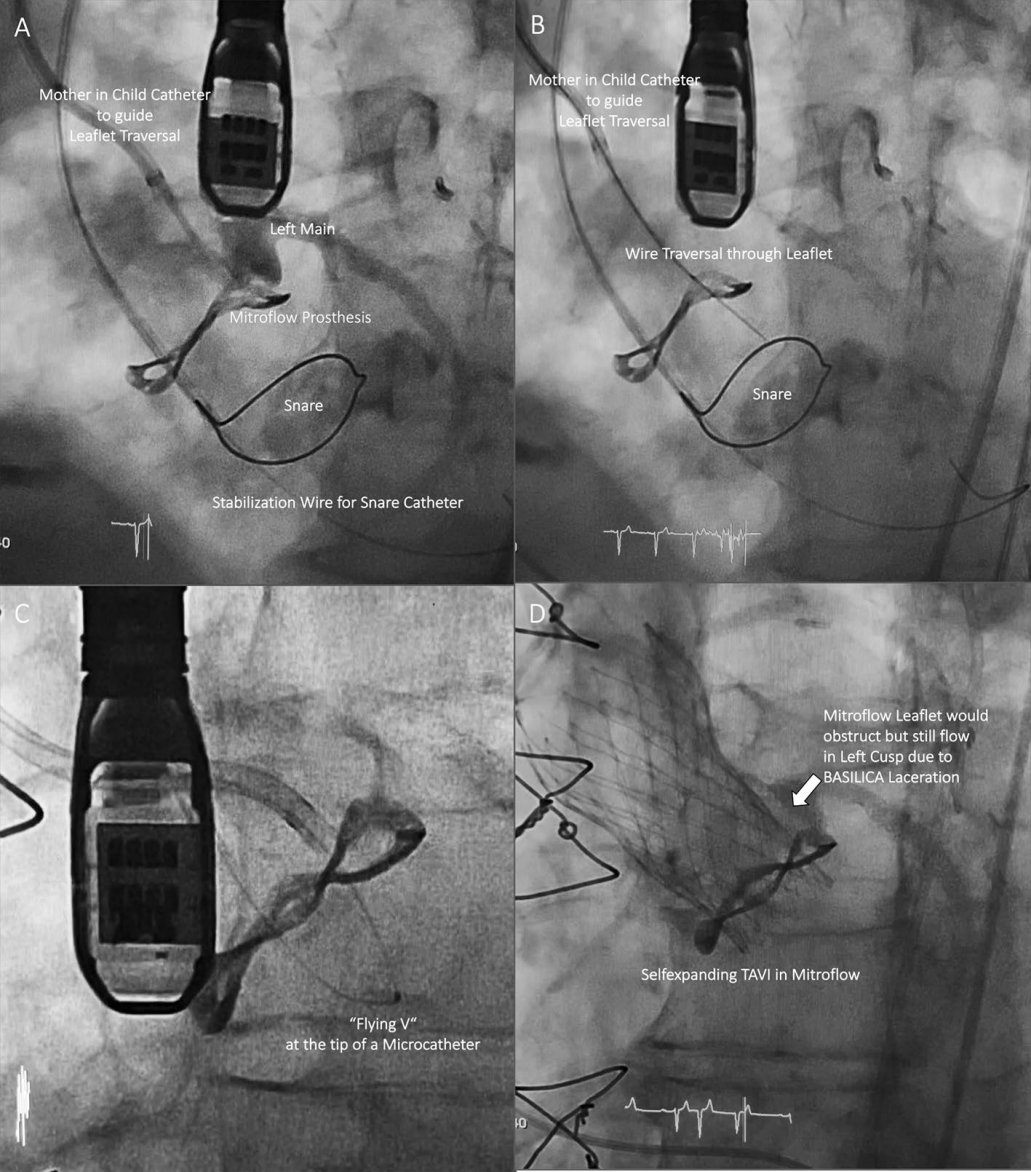
BASILICA (fluoroscopic images). **A** Catheter and wire setup before BASILICA. **B** Leaflet traversal into the snare. **C** Flying V configuration. **D** Result after successful leaflet laceration and THV implantation. *BASILICA* bioprosthetic or native aortic scallop intentional laceration to prevent iatrogenic coronary artery obstruction, *THV* transcatheter heart valve

The BASILICA procedure was performed under general anesthesia immediately prior to TAVI via bilateral femoral access. Correct positions and orientations of catheters and guidewires were checked and guided using transesophageal echocardiography and fluoroscopy using valvular front- and side-views pre-determined by MSCT before the procedure. Procedural setup routinely included insertion of a cerebral embolic protection device (Sentinel, Boston Scientific, Marlborough, MA, USA). The BASILICA technique has been described in detail elsewhere [6, 8, 9, 14]. In brief, two guiding catheters are positioned on either side of the aortic leaflet at risk, with a traversal 0.014-in. guidewire (Astato XS 20 300 cm, Asahi Intecc, Aichi, Japan) insulated in a micro-guide catheter (FineCross MG, Terumo, Tokyo, Japan) in the aortic root, and a snare (OneSnare, Merit Medical, South Jordan, UT, USA) in the left ventricular outflow tract (Fig. 4A). The electrified guidewire is directed through the base of the target leaflet into the snare (Fig. 4B). The guidewire is then externalized by snare retrieval forming a loop through the penetrated leaflet between the two guiding catheters (Fig. 4C). Intentional laceration of the target leaflet is achieved by focally applied electricity while gently pulling both free ends of the guidewire. Before splitting the leaflet, the guidewire is manually denuded of PTFE insulation at the mid-shaft to confine electrical contact with leaflet tissue, and kinked to form a *flying V* configuration. A standard electrosurgical generator (Covidien Force FX, Medtronic, Minneapolis, MN, USA) was used for electrical current supply. Implantation of the THV in the native or bioprosthetic aortic valve, respectively, was performed immediately afterwards as a standardized approach (Fig. 4D) [6, 8, 9, 14].

Supplementary file1 (MP4 141709 kb) Clip 1 Step-by-step guidance for BASILICA with fluoroscopic/echocardiographic images and ex vivo demonstration. BASILICA bioprosthetic or native aortic scallop intentional laceration to prevent iatrogenic coronary artery obstruction

### Statistical analysis

Data are presented as absolute numbers and percentages for categorical variables, and median values (25th percentile, 75th percentile) for continuous variables.

## Results

### Baseline characteristics

Patient baseline characteristics are depicted in Table 1. The study population (*n* = 15) was nearly equally distributed regarding gender (female: 53.3%) and characterized by an elderly patient cohort (81.0 [78.1, 84.4] years), with elevated rates of atrial fibrillation (53.3%), coexisting coronary artery disease (46.7%) and chronic kidney disease (60.0%) resulting in intermediate to high surgical risk according to established risk scores (EuroSCORE II: 10.6 [6.3, 14.8]%; STS PROM: 3.4 [2.4, 7.2]%).

**Table 1:** Patient baseline characteristics

Characteristics	(*n* = 15)
Age (years)	81.0 (78.1, 84.4)
Female gender [*n* (%)]	8 (53.3)
Body mass index (kg/m^2^)	26.0 (23.1, 28.2)
EuroSCORE II (%)	10.6 (6.3, 14.8)
STS PROM (%)	3.4 (2.4, 7.2)
NYHA functional class III or IV [*n* (%)]	12 (80.0)
Atrial fibrillation [*n* (%)]	8 (53.3)
Prior stroke [*n* (%)]	1 (6.7)
Peripheral artery disease [*n* (%)]	3 (20.0)
Chronic kidney disease (GFR < 50 mL/min) [*n* (%)]	9 (60.0)
Diabetes [*n* (%)]	2 (13.3)
COPD [*n* (%)]	3 (20.0)
Prior SAVR [*n* (%)]	13 (86.7)
Coronary artery disease [*n* (%)]	7 (46.7)
Prior myocardial infarction [*n* (%)]	2 (13.3)
Prior CABG [*n* (%)]	3 (20.0)
Prior PCI [*n* (%)]	4 (26.7)

Values are *n* (%) or median (interquartile range)

*CABG* coronary artery bypass graft, *COPD* chronic obstructive pulmonary disease, *GFR* glomerular filtration rate, *NYHA* New York Heart Association, *PCI*  percutaneous coronary intervention, *SAVR*   surgical aortic valve replacement, *STS PROM* Society of Thoracic Surgeons predicted risk of mortality

13 out of 15 patients (86.7%) had a history of previous bioprosthetic SAVR, three of them (23.1%) had undergone concomitant coronary artery bypass graft surgery. Surgical bioprostheses were stented prostheses with either internally mounted (Hancock II, Medtronic, *n* = 4, 30.8%) or externally mounted leaflets (Mitroflow, Sorin, *n* = 5, 38.5%; Trifecta, St. Jude Medical/Abbott, *n* = 3, 23.1%), and one stentless prosthesis (Freedom Solo, Sorin, 7.7%). Morphologies of bioprosthetic valve degeneration were severe stenosis in 5 patients (33.3%), severe regurgitation in 6 patients (40.0%) and combined stenosis/regurgitation in 2 patients (13.3%). Furthermore, 2 patients (13.3%) with severe native valve AS were treated. Detailed pre-procedural data are presented in Table 2.

**Table 2:** Pre-procedural data

Types of surgical bioprostheses (*n* = 13)	
Stented prostheses, internally mounted leaflets	
Hancock II, Medtronic	4 (30.8)
Stented prostheses, externally mounted leaflets	
Mitroflow, Sorin	5 (38.5)
Trifecta, St. Jude Medical/Abbott	3 (23.1)
Stentless prosthesis	
Freedom Solo, Sorin	1 (7.7)
Morphology of aortic valve degeneration (*n* = 15)	
Native valve aortic stenosis	2 (13.3)
Surgical bioprostheses	
Aortic stenosis	5 (33.3)
Aortic regurgitation	6 (40.0)
Combined stenosis/regurgitation	2 (13.3)
Echocardiographic parameters (*n* = 15)	
Mean AV gradient (mmHg)	28.0 (19.0, 38.0)
Effective orifice area (cm^2^)	0.9 (0.8, 1.2)
Mitral regurgitation ≥ moderate	4 (26.7)
Tricuspid regurgitation ≥ moderate	4 (26.7)
LVEF (%)	55.0 (44.0, 60.0)

Values are *n* (%) or median (interquartile range)

*AV* aortic valve, *LVEF* left ventricular ejection fraction

### Indication for BASILICA

MSCT-based parameters utilized for risk assessment of coronary obstruction by left coronary cusp (LCC) and/or right coronary cusp (RCC) are summarized in Table 3. Out of 8 patients (53.3%) presenting with Deficient Sinus, high risk for LCC obstruction was found in 6 patients (40.0%) with low LCA height (5.2 [3.7, 9.8] mm) and small LCC SOV diameter (27.3 [26.3, 29.3] mm) resulting in low VTC distance to LCA (2.9 [2.2, 3.4] mm). In three patients (20.0%) risk for RCC obstruction was identified, with low RCA height (10.1 [9.0, 10.2] mm), small RCC SOV diameter (25.8 [24.2, 26.6] mm) and low VTC distance to RCA (2.8 [2.5, 2.9] mm). Of these, 1 patient (6.7%) was at risk for obstruction of both coronary ostia. Consequently, BASILICA was attempted for single LCC in 5 patients (33.3%), for single RCC in 2 patients (13.3%), and for both cusps in 1 patient (6.7%), respectively.

**Table 3:** MSCT-based risk assessment of coronary obstruction

Deficient Sinus (*n* = 8)^a^	
LCC (*n* = 6)	
LCA height (mm)	5.2 (3.7, 9.8)
SOV diameter (LCC) (mm)	27.3 (26.3, 29.3)
VTC (LCA) (mm)	2.9 (2.2, 3.4)
RCC (*n* = 3)	
RCA height (mm)	10.1 (9.0, 10.2)
SOV diameter (RCC) (mm)	25.8 (24.2, 26.6)
VTC (RCA) (mm)	2.8 (2.5, 2.9)
Sequestered Sinus (*n* = 7)	
LCC (*n* = 7)	
STJ height (mm)	16.6 (14.8, 18.3)
STJ diameter (mm)	28.3 (26.5, 29.4)
VTSTJ (mm)	2.5 (0.6, 2.9)
VTSTJ = 0 mm [*n* (%)]	2 (28.6)

Values are *n* (%) or median (interquartile range)

*LCA* left coronary artery, *LCC* left coronary cusp, *RCA* right coronary artery, *RCC* right coronary cusp, *SOV* sinus of Valsalva, *STJ* sinotubular junction, *VTC* valve-to-coronary distance, *VTSTJ* valve-to-sinotubular junction distance

^a^In one patient, BASILICA was performed for both LCC and RCC

Further, 7 patients (46.7%) were identified with Sequestered Sinus, all at high risk for LCC obstruction, characterized by low STJ height (16.6 [14.8, 18.3] mm) in combination with low STJ diameter (28.3 [26.5, 29.4] mm) and resulting in low VTSTJ distance (2.6 [2.5, 3.1] mm). Therefore, BASILICA was attempted for single LCC in these patients. Overall, BASILICA was intended for 16 leaflets (LCC: *n* = 13, 81.3%; RCC: *n* = 3, 18.8%) in 15 patients.

### Procedural and VARC-2 outcomes

**Fig. 5:**
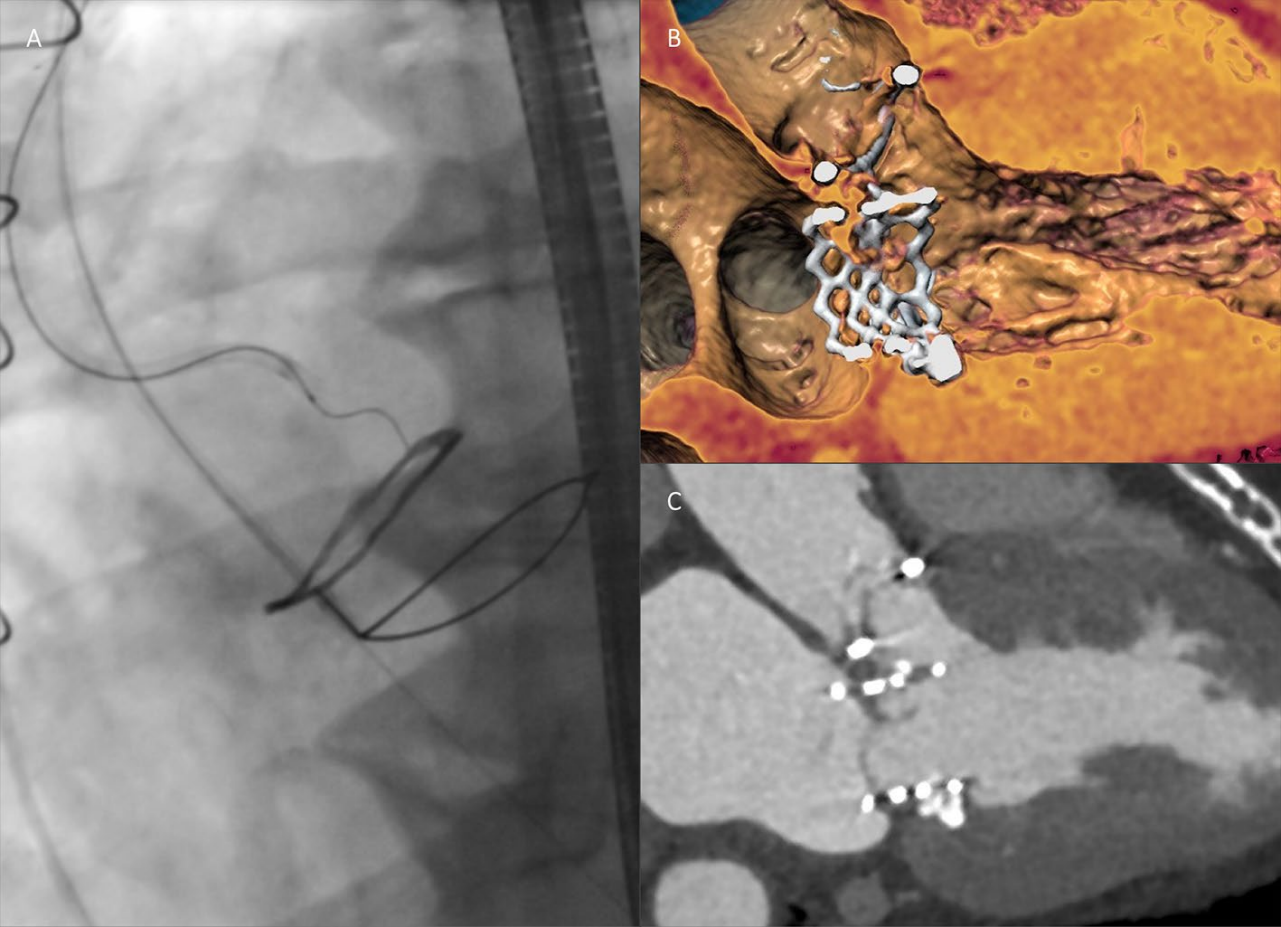
BASILICA failure. **A** Impenetrable calcified leaflet (Sorin Mitroflow surgical valve). **B** Wire-interference with a THV in mitral position (Sapien-in-MAC), Sorin Mitroflow aortic valve, pre-TAVI three-dimensional CT reconstruction. **C** pre-TAVI CT (see **B**). *BASILICA* bioprosthetic or native aortic scallop intentional laceration to prevent iatrogenic coronary artery obstruction, *CT* computed tomography, *MAC* mitral annulus calcification, *TAVI* transcatheter aortic valve implantation, *THV* transcatheter heart valve

Detailed procedural data are given in Table 4. BASILICA was attempted for single LCC in 12 patients (80.0%), for single RCC in 2 patients (13.3%), and for both cusps in 1 patient (6.7%), respectively. Technical success of BASILICA was achieved in 13 out of 15 patients (86.7%). In 2 patients (13.3%) with single LCC and single RCC attempts, respectively, leaflet traversal could not be performed successfully. Leaflet laceration was successful in all leaflets traversed. Reasons for traversal failure were impenetrable leaflet calcification of an externally mounted bioprosthesis in one case (Fig. 5A) and interference with a formerly implanted THV in mitral valve position (Fig. 5B, C) in another case.

**Table 4:** Procedural data

Characteristics	
Target leaflet for BASILICA	
LCC	12 (80.0)
RCC	2 (13.3)
Both cusps	1 (6.7)
Cerebral protection device	15 (100)
Transfemoral access for TAVI	15 (100)
Balloon pre-dilatation	0 (0)
Balloon post-dilatation	5 (33.3)
Bioprosthetic valve fracture	1 (6.7)
Total procedure time (min)	160 (146, 178)
Fluoroscopy time (min)	52 (45, 56)
Contrast agent volume (mL)	281 (172, 352)
THV prostheses	
Self-expanding THV	
CoreValve Evolut R, Medtronic	14 (93.3)
23 mm/26 mm	5 (33.3)/9 (60.0)
Balloon-expandable THV	
Sapien 3 Ultra 26 mm, Edwards Lifesciences	1 (6.7)

Values are *n* (%) or median (interquartile range)

*BASILICA* bioprosthetic or native aortic scallop intentional laceration to prevent iatrogenic coronary artery obstruction, *LCC* left coronary cusp, *RCC* right coronary cusp, *TAVI* transcatheter aortic valve implantation, *THV* transcatheter heart valve

**Fig. 6:**
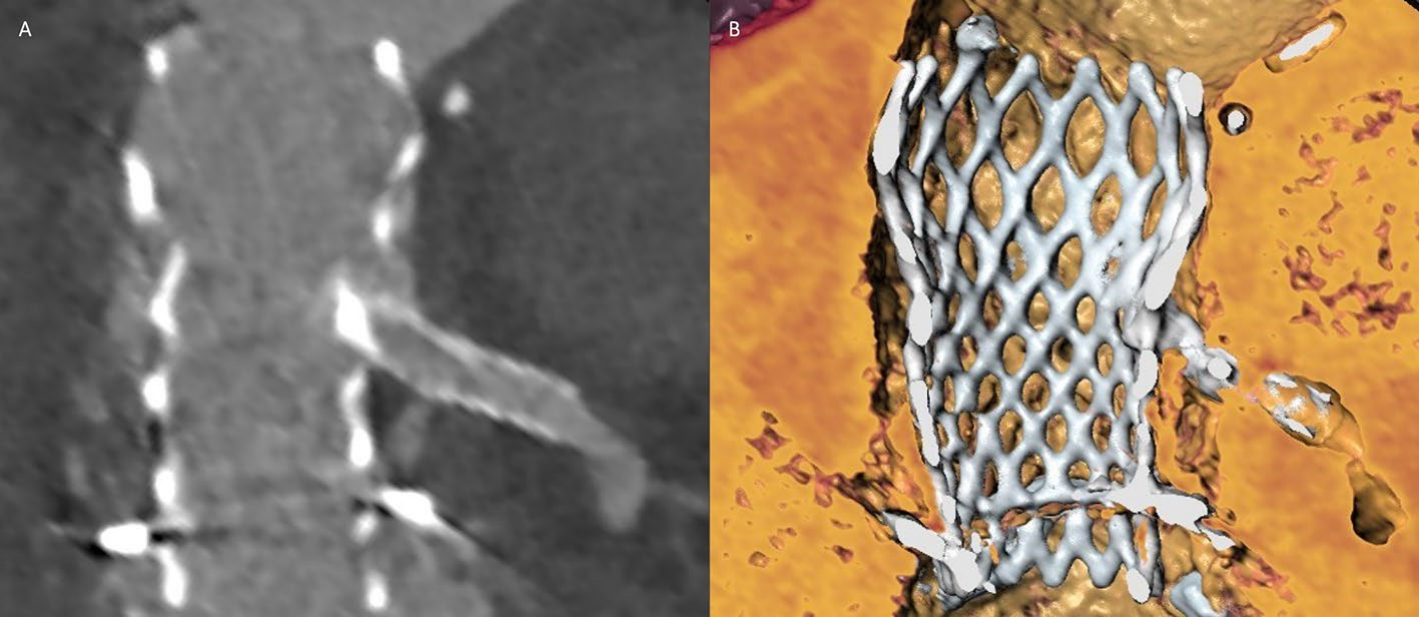
Chimney stenting. **A** Chimney stent implantation in left coronary artery after BASILICA failure (CoreValve Evolut R in Sorin Mitroflow), post-TAVI CT. **B** post-TAVI three-dimensional CT reconstruction (see **A**). *BASILICA* bioprosthetic or native aortic scallop intentional laceration to prevent iatrogenic coronary artery obstruction, *CT* computed tomography, *TAVI* transcatheter aortic valve implantation

In both patients, coronary stents were pre-positioned of which one was deployed after TAVI due to the probability of obstruction on the height of STJ (Fig. 6) and the other removed due to sufficient coronary flow. Hemodynamic instability did not occur in any procedure during the BASILICA maneuver.

**Fig. 7:**
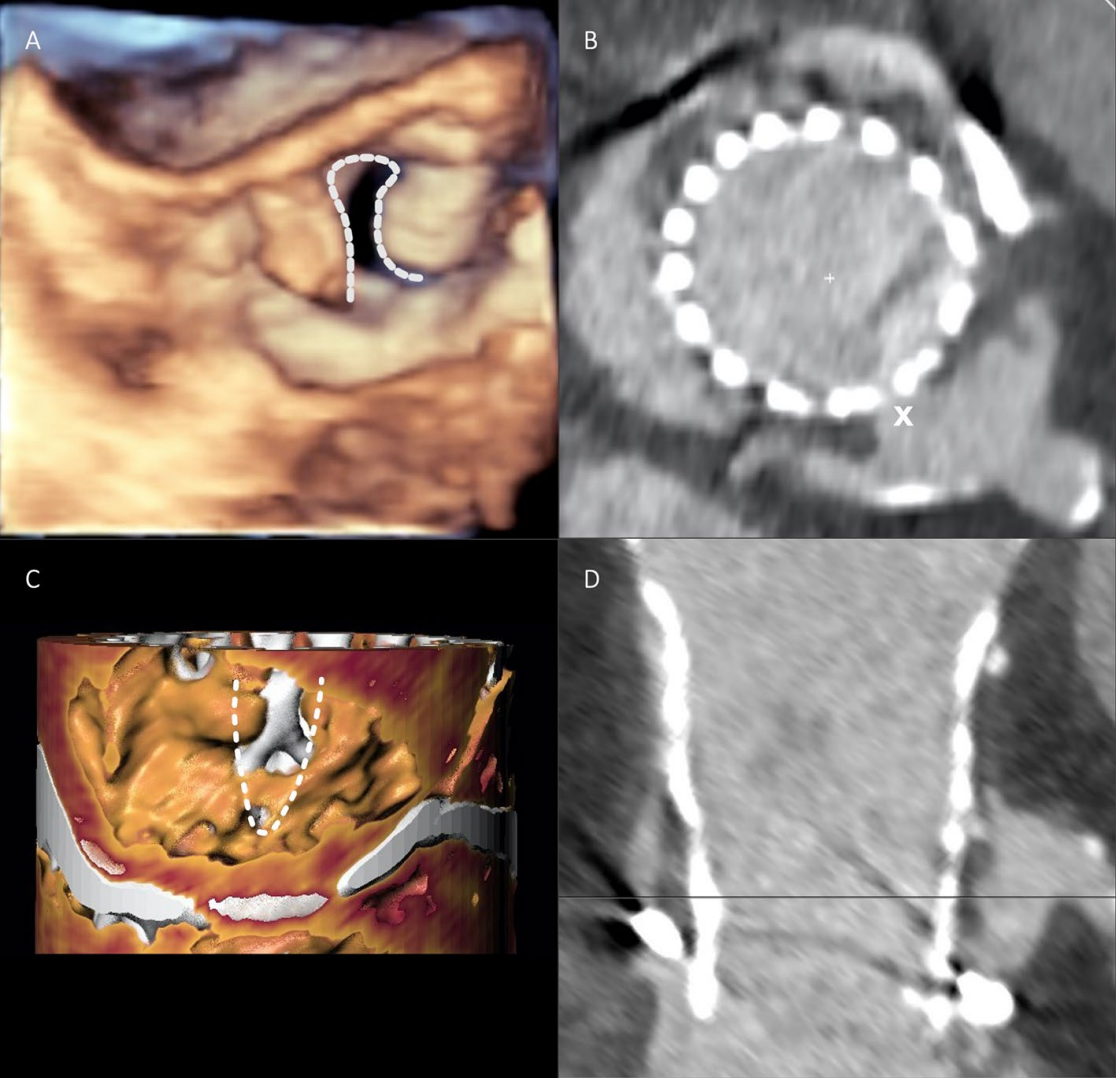
Successful BASILICA. **A** Confirmation of successful leaflet laceration by peri-procedural transesophageal echocardiography. **B** Lacerated leaflet (x) in a patient with Sequestered Sinus, post-TAVI CT. **C** Confirmation of lacerated leaflet (dashed line), post-TAVI three-dimensional CT reconstruction. **D** Post-TAVI CT in a patient with Sequestered Sinus (CoreValve Evolut R in Sorin Mitroflow) after BASILICA. *BASILICA* bioprosthetic or native aortic scallop intentional laceration to prevent iatrogenic coronary artery obstruction, *CT* computed tomography, *TAVI* transcatheter aortic valve implantation

THV implantation immediately after the BASILICA procedure was successful in all 15 patients. Transfemoral access for TAVI was chosen in all cases. In 14 patients (93.3%), a self-expanding CoreValve Evolut R (Medtronic) was implanted. In 1 patient (6.7%), a balloon-expandable Sapien 3 Ultra THV (Edwards Lifesciences) was used. Intentional bioprosthetic valve fracture by high-pressure balloon inflation following TAVI was performed in one case (6.7%) due to increased transvalvular gradient post-implant. Leaflet laceration was confirmed by transesophageal echocardiography during the procedure (Fig. 7A) and by post-TAVI MSCT (Fig. 7B, C) in selected patients.

Adjudicated VARC-2 efficacy and safety endpoints are depicted in Table 5. No all-cause mortality, myocardial infarction, stroke or acute kidney injury was documented until 30 days. There were no cases requiring new permanent pacemaker implantation or repeat procedure due to valve-related dysfunction. A major access site complication with resultant major bleeding on postoperative day one occurred in 1 patient (6.7%).

**Table 5:** VARC-2 efficacy and safety endpoints

Technical success (intra-procedure)	13 (86.7)
Procedural mortality	0 (0)
Successful leaflet traversal and laceration	13 (86.7)
Successful implantation of the cerebral protection device	15 (100)
Coronary obstruction/intervention at target leaflet	1 (6.7)
Successful first TAVI	15 (100)
Emergency surgery or reintervention related to BASILICA/TAVI	0 (0)
Early safety (at 30 days)	13 (86.7)
All-cause mortality	0 (0)
Myocardial infarction	0 (0)
Coronary obstruction requiring intervention	1 (6.7)
Stroke	0 (0)
Major vascular/access site complication	1 (6.7)
Life-threatening bleeding (BARC type ≥ 3b)	0 (0)
Acute kidney injury (AKIN stage 2 or 3)	0 (0)
Cardiac tamponade	0 (0)
Major cardiac structural complication	0 (0)
New permanent pacemaker implantation	0 (0)
Valve-related dysfunction requiring repeat procedure	0 (0)
THV function at discharge	
Paravalvular leakage > trace	0 (0.0)
Mean AV gradient, mmHg	9.0 (5.5, 14.0)
Mean AV gradient > 20 mmHg	2 (13.3)

Values are *n* (%) or median (interquartile range)

*AKIN* Acute Kidney Injury Network, *AV* aortic valve, *BARC* Bleeding Academic Research Consortium, *BASILICA* bioprosthetic or native aortic scallop intentional laceration to prevent iatrogenic coronary artery obstruction, *TAVI* transcatheter aortic valve implantation, *THV* transcatheter heart valve, *VARC-2* Valve Academic Research Consortium-2

Echocardiography at discharge revealed sufficient valvular function in all patients. Mean transprosthetic gradient was 9.0 (5.5, 14.0) mmHg. In 2 patients (13.3%) increased mean aortic valve gradient > 20 mmHg was measured at discharge echocardiography. No paravalvular leakage greater than trace was observed.

No statistically significant differences in procedure time, dose area product or contrast agent were found comparing the first half of patients successfully treated with a single leaflet laceration (*N* = 11) to the second half. However, procedure time (156.6 ± 12.5 min vs. 149.2 ± 25.6 min, *p* = 0.55) and dose area product (17,514.8 ± 6775.7 cGy cm^2^ vs. 13,798.2 ± 4248.2 cGy cm^2^, *p* = 0.35) numerically decreased, while usage of contrast agent numerically increased over time (192.6 ± 87.8 mL vs. 234.2 ± 111.0 mL, *p* = 0.57).

## Discussion

We herein present our single-center experience with the BASILICA technique to facilitate TAVI procedures in patients at high risk of coronary obstruction. While being safe and feasible future trials need to evaluate detailed inclusion criteria prospectively.

### Risk assessment of iatrogenic coronary obstruction

Recently, several studies have assessed the incidence and predictors of coronary obstruction in patients undergoing native valve TAVI or ViV TAVI for degenerated surgical bioprostheses. The incidence of coronary obstruction in patients treated for native AS appears to be low, with low coronary height and small SOV diameters, defined as Deficient Sinus, causing direct occlusion of a coronary artery by native leaflet [4, 15, 16]. A coronary height cut-off of 12 mm has been established as an indicator of high risk for direct coronary obstruction [4, 17]. In a large multicenter registry of 6688 patients, who underwent TAVI or ViV TAVI, rates of coronary obstruction were 0.62% and 2.48% for TAVI in native AS and ViV-TAVI, respectively, indicating a higher risk of coronary obstruction in patients with prior SAVR [4]. Indeed, ViV TAVI was identified as an independent predictor of coronary obstruction in this cohort [4].

In patients undergoing ViV TAVI, two mechanisms potentially causing coronary obstruction have to be distinguished. The primary mechanism is direct occlusion of a coronary artery by a prosthetic leaflet in patients with Deficient Sinus [17]. A second mechanism is premised on the extension of bioprosthetic structures to the STJ, in particular in the presence of low STJ height and small STJ diameter, resulting in indirect coronary obstruction by the creation of a covered cylinder after THV implantation (Sequestered Sinus) [17]. Besides these anatomical risk determinants, bioprosthetic valve characteristics like supra-annular leaflet position, high leaflet profile and externally mounted leaflets in stented prostheses are considered for risk assessment before ViV TAVI [18]. In an analysis of 1612 patients enrolled in the *Valve-in-Valve International Data* (VIVID) Registry, Ribeiro et al. observed significantly higher rates of coronary obstruction in patients with stentless bioprostheses (3.7%) and stented prostheses with externally mounted leaflets (6.4%) compared to those with internally mounted leaflets (0.7%) [2]. In addition, VTC distance was identified as a valid and independent MSCT-derived predictor of coronary obstruction [2]. Recently, Tang et al. proposed an algorithm for risk assessment of coronary obstruction before ViV TAVI based on VTC and VTSTJ cut-off values of < 4 mm and < 2.5–3.5 mm, respectively. The authors also introduced the VIVID classification, which specifies aortic root anatomies based on the concept of Deficient and Sequestered Sinus [19].

The above findings are supported by our own results in this single-center study, with most patients identified at high risk of coronary obstruction having a history of prior SAVR, and only two patients presenting with severe native AS. Moreover, more than half of the patients scheduled for ViV TAVI had either stented surgical bioprostheses with externally mounted leaflets or a stentless prosthesis. High risk of coronary obstruction in the present patient cohort was confirmed by low VTC and VTSTJ distances in the groups of Deficient and Sequestered Sinus, respectively. Considering all subjects, the LCA was identified as coronary artery at risk in the vast majority of cases, while the RCA was considered vulnerable in only two patients. This is consistent with findings from the above-mentioned multicenter registries, with LCA obstruction rates of 88.6% and 72.2%, respectively, referred to all coronary obstruction cases in native valve TAVI or ViV TAVI procedures [2, 4].

Chimney stenting represents an established bailout strategy for iatrogenic coronary occlusion after TAVI. By upfront positioning of a coronary guidewire, balloon or undeployed stent in the coronary artery at risk, a stent can be deployed between the displaced leaflets and the aortic wall in case of compromised coronary blood flow [7]. However, mechanical stent compression, restenosis and late stent thrombosis have to be taken into account when using this technique. Moreover, coronary re-access after chimney stenting is extremely challenging [7].

### BASILICA technique

The concept of the BASILICA procedure for the prevention of coronary obstruction during TAVI was first described in 2018 by Khan et al. in a series of compassionate use cases [8]. Since then, feasibility, efficacy and safety of this novel technique have been demonstrated by few studies [9, 10]. One large series from a multicenter registry comprised 30 patients, in whom BASILICA was intended during native valve TAVI and ViV TAVI. Successful traversal and laceration were achieved in 93.3% of these cases with the absence of procedural coronary obstruction or procedural mortality. Rates of cardiovascular mortality (3.3%), disabling stroke (3.3%) and life-threatening bleeding (6.6%) were low, with an elevated rate of major vascular complications (20.0%). Recently, the largest retrospective study to date was published including 214 patients from 25 centers. Again, cardiovascular mortality was low with 2.8% and stroke risk being 2.8% [20]. The results of the present study are in line with these data demonstrating solid feasibility (successful in 92.3% of LCC and 66.7% of RCC intents) and safety of the procedure without procedural mortality or valve-related dysfunction requiring repeat procedure. Rates of vascular or hemorrhagic complications were low in this single-center series. Stroke or other neurological deficits were not observed in this small patient cohort. One might expect increased risk of calcific embolization during BASILICA maneuver due to prolonged catheter manipulation along the target cusp and leaflet laceration debris. Routine insertion of a cerebral embolic protection device in all subjects potentially may have accounted for the absence of neurological events. In contrast, Khan et al. described three cases of stroke in 30 subjects enrolled in their BASILICA trial, with the use of a cerebral embolic protection device in merely one of them [9]. However, in the large cohort described above embolic protection was not associated with differences in stroke rate [20]. Therefore, specific impact of BASILICA on the risk of stroke during TAVI remains uncertain until larger investigative series are available. Until then, we would advocate for a liberal use of embolic protection devices for this complex subset of patients.

### Transesophageal echocardiography guidance

Optimal intra-procedural visualization with delineation of critical cardiac structures is crucial for a safe and successful BASILICA procedure. Utilization of both fluoroscopic front and side view projections of the target cusp enabling to implement of precise leaflet traversal site and direction is recommended by its pioneering experts [6]. However, the feasibility of achieving fluoroscopic projections pre-determined by MSCT before the procedure in a standard catheter laboratory is limited in most cases [21]. In particular, LCC front view and RCC side view projections can be challenging to obtain or are unachievable. Thus, the usage of modified projections not perfectly anatomically aligned or repositioning of the patient on C-arm table has been proposed. Transesophageal echocardiography allows for a visualization of the key steps and structures, including LCA and RCA ostia, during the entire leaflet traversal scenario. This can be helpful since coronary ostia occasionally have an eccentric origin in relation to the center of the target cusp affecting the target point for leaflet traversal and laceration [22]. Therefore, we decided to use intra-procedural transesophageal echocardiography as an additional and important guidance-imaging tool to facilitate and shorten the procedure. This is currently also recommended by others [23]. Certainly, implementation by an experienced echocardiographer is mandatory for safe and reliable echocardiographic guiding and validation of the BASILICA procedure.

### Limitations

This study is limited by its study design and small sample size, and may, therefore, only serve to generate hypotheses. However, the presented results with this novel technique are in line with previous studies of comparable sample sizes and support the broader use of this procedure in patients at risk. The results of the prospective BASILICA trial (NCT03381989) are highly awaited and will shed more light on outcomes of patients at high risk for coronary obstruction undergoing native valve TAVI or ViV TAVI.

## Conclusions

The results of our present single-center study support the use of the BASILICA technique as a safe and effective preventive treatment option in patients at high or prohibitive risk of iatrogenic coronary obstruction during native valve TAVI and ViV TAVI. The procedure was performed successfully in the majority of patients with absence or low rates of VARC-2 defined peri- and post-procedural complications.

## Supplementary Information

Below is the link to the electronic supplementary material.
