# Correction to: Prevention of coronary obstruction in patients at risk undergoing transcatheter aortic valve implantation: the Hamburg BASILICA experience

**DOI:** 10.1007/s00392-021-01904-0

**Published:** 2021-08-04

**Authors:** Dirk Westermann, Sebastian Ludwig, Daniel Kalbacher, Clemens Spink, Matthias Linder, Oliver D. Bhadra, Julius Nikorowitsch, Lara Waldschmidt, Till Demal, Lisa Voigtländer, Andreas Schaefer, Moritz Seiffert, Simon Pecha, Niklas Schofer, Adam B. Greenbaum, Hermann Reichenspurner, Stefan Blankenberg, Lenard Conradi, Johannes Schirmer

**Affiliations:** 1 Department of Cardiology, University Heart and Vascular Center Hamburg, University Medical Center Hamburg-Eppendorf, Martinistrasse 52, 20246 Hamburg, Germany; 2 German Center for Cardiovascular Research (DZHK), Partner Site Hamburg/Luebeck/Kiel, Hamburg, Germany; 3 Department of Diagnostic and Interventional Radiology and Nuclear Medicine, University Medical Center Hamburg-Eppendorf, Hamburg, Germany; 4 Department of Cardiovascular Surgery, University Heart and Vascular Center Hamburg, Hamburg, Germany; 5 Structural Heart and Valve Center, Emory University Hospital, Atlanta, GA USA

## Correction to: Clinical Research in Cardiology 10.1007/s00392-021-01881-4

The original version of this article, published on June 22, 2021, contained a mistake. The affiliations were assigned incorrectly. The correct information is given above. The original article has been corrected.

